# Frequency-Risk and Duration-Risk Relationships between Aspirin Use and Gastric Cancer: A Systematic Review and Meta-Analysis

**DOI:** 10.1371/journal.pone.0071522

**Published:** 2013-07-30

**Authors:** Xiaohua Ye, Jinjian Fu, Yi Yang, Yanhui Gao, Li Liu, Sidong Chen

**Affiliations:** 1 School of Public Health, Guangdong Key Laboratory of Molecular Epidemiology, Guangdong Pharmaceutical University, Guangzhou, Guangdong, China; 2 School of Public Health and Tropical Medicine, Southern Medical University, Guangzhou, Guangdong, China; 3 Liuzhou Municipal Maternity and Child Healthcare Hospital, Liuzhou, Guangxi, China; Enzo Life Sciences, Inc., United States of America

## Abstract

**Background:**

Although previous meta-analyses have suggested an association between aspirin use and risk of gastric cancer, current evidence is inconsistent. Additionally, it remains unclear whether there are frequency-risk and duration-risk relationships and if a threshold of effect exists.

**Methods:**

We identified studies by searching MEDLINE and PUBMED databases and reviewing relevant articles. We derived the summary risk estimates using fixed-effects or random-effects model based on homogeneity analysis. The dose-response meta-analysis was performed by linear trend regression and restricted cubic spline regression. Potential heterogeneity was tested using the *Q* statistic and quantified with the *I* ^2^ statistic. Subgroup analyses and Galbraith plots were used to explore the potential sources of heterogeneity. Publication bias was evaluated with funnel plots and quantified by the Begg's and Egger's test.

**Results:**

Fifteen studies were included in this meta-analysis. There was an overall 29% reduced risk of gastric cancer corresponding to aspirin use (RR  = 0.71, 95% CI 0.60–0.82). We found there are nonlinear frequency-risk and linear duration-risk relations between aspirin use and gastric cancer. A monotonically decreasing relation was observed only for low-frequency (≤4.5 times/week) aspirin intake (10% decreased risk for once/week, 19% for twice/week and 29% for 4.5 times/week), and the frequency threshold of aspirin use is 4.5 times per week. Regarding those with duration of aspirin use, there was a tendency towards stronger risk reduction of gastric cancer for longer aspirin use (10% decreased risk for 4 years, 19% for 8 years and 28% for 12 years), and no duration threshold was observed.

**Conclusion:**

Our findings suggest that long-term (≥4 years) and low-frequency (1–4.5 times per week) aspirin use is associated with a statistically significant, dose-dependent reduction in the risk of gastric cancer.

## Introduction

Until the mid-1990s, gastric cancer has been the most common cause of cancer deaths worldwide [1]. Although rates have been gradually declining in recent decades and gastric cancer has become a relatively rare cancer in North America and most parts of Africa [2], it remains prevalent in Eastern Asia, Eastern Europe, and South America. Therefore, gastric cancer remains the fourth most common cancer and the second most common cause of cancer deaths worldwide, as of 2008 [2], [3]. It is well known that earlier diagnosis of gastric cancer can effectively improve prognosis, but the disease is often clinically silent at an early stage, and in most countries, patients have advanced stages at diagnosis [4]. In addition, the all-stages 5-year relative survival rate is only 26% in white Americans and 27% in African Americans [5]. Therefore, primary prevention of gastric cancer is extremely important for public health.

Gastric carcinogenesis is a multi-step and multi-factorial process, although its etiology is not fully understood. Several studies [6]–[11] have shown that aspirin and other nonsteroidal anti-inﬂammatory drugs (NSAIDs) have been associated with a reduced risk of gastric cancer. The chemopreventive effect of NSAIDs has been attributed to their inhibition of cyclooxygenase (COX)-2, the enzymes responsible for the synthesis of prostaglandins. COX-2 has been reported to be overexpressed in several gastrointestinal malignancies, including gastric cancer, and participates in several key cellular activities, such as cell proliferation, apoptosis, and angiogenesis [12], [13]. Some studies suggest the existence of other anticarcinogenic mechanisms of NSAIDs, such as the induction of apoptosis through COX-independent pathways and the up-regulation of tumor suppression genes [14]–[16].

A few quantitative reviews of epidemiological studies reported an inverse association between aspirin use and gastric cancer [6]–[10], while another meta-analysis found no significant association with aspirin use [11]. The inconsistencies of the reports could be attributed to several factors including age, sex, race, socioeconomic status, study design, sites of cancer, sample sources, and geographical regions. Therefore, it is necessary to adjust for these confounding factors when assessing the risk ratio (RR) or the odds ratio (OR) for aspirin use and gastric cancer. In addition, none of the previous quantitative reviews focused on the frequency-risk and duration-risk relationships between aspirin use and gastric cancer risk. In this study, we systematically identified case-control and cohort studies on the issue published up to February 2013. We then carried out a dose-response meta-analysis to evaluate the threshold effect between aspirin intake and the risk of gastric cancer, so as to guide rational use of aspirin as a chemopreventive agent against gastric cancer.

## Methods

### Search Strategy

The meta-analysis was conducted following the PRISMA guidelines and the PRISMA checklist was listed in Table S1 [17]. We searched MEDLINE and PUBMED, from January 1980 to February 2013, with the following searching terms: [aspirin OR NSAID OR ‘nonsteroidal anti-inﬂammatory drugs’] AND [‘gastric cancer’ OR ‘stomach cancer’ OR ‘gastric neoplasm’ OR ‘stomach neoplasm’ OR‘gastric carcinoma’ OR ‘stomach carcinoma’]. In addition, reference lists of all retrieved articles and previous systematic reviews were checked for further eligible publications. We restricted our search to studies performed in human studies and published in English.

### Inclusion and Exclusion Criteria

Two reviewers (XH Ye and JJ Fu) independently identified articles eligible for in-depth examination using the following inclusion and exclusion criteria. The inclusion criteria required studies to: (i) have a case-control, cohort or randomized controlled trial (RCT) study design; (ii) provide information on aspirin use in relation to gastric cancer considered separately from other NSAIDs; and (iii) report an estimate of association such as RR and their 95% confidence intervals (CIs), or enough information to compute them. Studies were excluded if: (i) studies were cross-sectional surveys, case reports, review articles, editorials, and clinical guidelines; (ii) they were done in populations with specific precancerous diseases (eg, adenomas) and rheumatoid arthritis. When multiple articles reported the same study population, we included only the most recent and informative publication that met the inclusion criteria. Any discrepancies on articles meriting inclusion between reviewers were resolved by a consensus meeting of three authors (XH Ye, JJ Fu, and SD Chen).

### Data Extraction

Two investigators (XH Ye and JJ Fu) reviewed and extracted data independently by using a standardized form, and then cross-checked the data together. Disagreements were resolved by consensus. For each study, we extracted information on the first author's name, study location, publication year, study design, sample sources, number of subjects, site of cancer, adjusted factors, definition of aspirin use, frequency and duration of aspirin use, diagnosis method, RR (approximated by OR for case-control studies) and the corresponding 95% CI for regular aspirin use or alternatively any use. Throughout this paper, RR is used to refer to all risk estimates including ORs and HRs.

### Statistical analysis

Heterogeneity among studies was tested using the Cochrane Q statistic (significant at *P*<0.1) and quantified with the *I*2 statistic, which describes the variation of influence that is attributable to heterogeneity across studies [18], [19]. Subgroup analyses were performed according to study designs (case-control, cohort or RCT), sites of cancer (cardia or noncardia), sample sources (population-based or hospital-based), geographical region (USA, Europe, and Asia), *helicobacter pylori* (*H. pylori*) infection (yes or no) and adjustments for covariates, so as to explore the source of heterogeneity. Galbraith plots were used to visualize the impact of individual studies on the overall homogeneity [18]. In the absence of individual heterogeneity, we could expect all the points to lie within the confidence bounds.

The presence and effect of publication bias were evaluated by visual inspection of Begg's funnel plot and tested by the Begg's test and Egger's test (significant at *P*<0.1) [19], [20]. Additionally, the trim-and-fill method was used to adjust the risk estimates when the tests for publication bias were statistically significant [21].

All relative risks were pooled by either fixed-effects model or random-effects model, depending on the overall heterogeneity among studies (fixed if *P*>0.1, random if *P*≤0.1). To derive the frequency-risk and duration-risk relationships between aspirin use and gastric cancer, we carried out stratified analysis and dose-response analysis on frequency and duration of aspirin use. The dose-response meta-analyses were carried out using linear trend regression and restricted cubic spline regression, choosing the best-fitting model [22], [23]. This analysis used data including the RRs and the corresponding 95%CI, number of cases and non-cases, and median of aspirin consumption levels for each comparison group. When intervals of aspirin categories were reported, the midpoint of the interval was chosen. For the open-ended upper interval, we used 1.2-fold its lower limit [24].

All statistical analyses were performed using Stata statistical software version 10.0. The metan, metabias, metafunnel, metatrim, and galbr commands were used for meta-analytic procedures (Command S1). In addition, the rc_spline command was used to create spline covariates and glst command was used to fit the linear or non-linear dose-response models (Command S1).

## Results

### Characteristics of Studies

**Figure 1:**
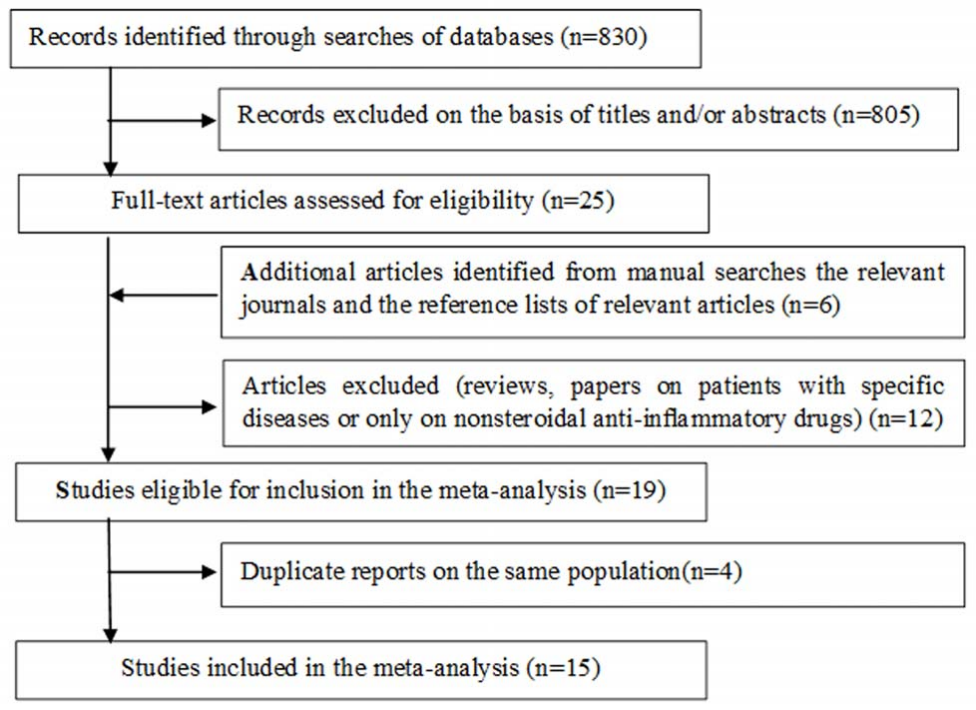
Flowchart of literature search and study selection.

The literature search and study selection process are shown in Figure 1. We initially identified 830 potentially relevant studies. Based on the scanning of the titles and abstracts, 805 articles were excluded. After reading the full text of the remaining studies and excluding 4 duplicate reports [25]–[28], 15 studies [10], [29]–[42] were included in the final analysis. The studies included 8 case-control studies [29]–[36] on a total of 4437 cases, 5 cohort studies [10], [37]–[40] on a total of 2340 cases and 2 RCT studies [41], [42] on a total of 91 cases. Nine of these studies were conducted in USA [10], [33]–[36], [38]–[41], while 5 were in Europe [30]–[32], [37], [42] and only one in Asia [29]. The main characteristics and findings of studies on aspirin and the risk of gastric cancer are given in Table S2(ever use versus nonuse), Table S3(frequency of use),and Table S4(duration of use).

### Ever use versus nonuse of aspirin use

**Figure 2:**
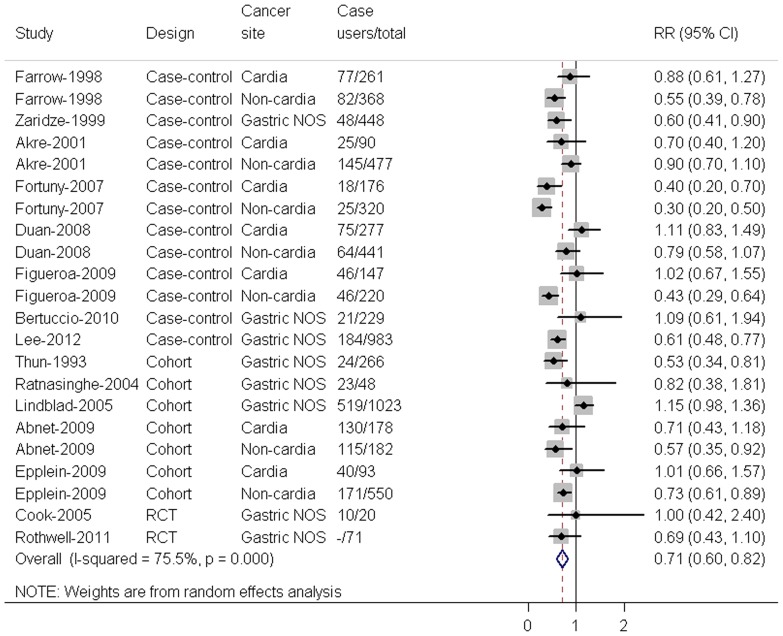
Forest plot for the association between aspirin use(ever use vs. nonuse) and risk of gastric cancer. The combined relative risk was achieved using random-effects model. Grey square represents relative risk in each study, with square size reflecting the study-specific weight and the 95% CI represented by horizontal bars. The diamond indicates summary risk estimate. Gastric NOS means that the location of the tumors within the stomach was not specified.

The overall RR for gastric cancer for aspirin use was 0.71(95% CI 0.60–0.82), and some heterogeneity was observed (I^2^  = 75.5%, P for heterogeneity = 0.000; Figure 2). We carried out stratified analyses to assess the heterogeneity across subgroups defined by study design, cancer site, sample source, geographical region, and *H. pylori* infection (Table 1). The estimates obtained did not substantially differ from the overall ones and no significant heterogeneity was found for any of the stratification variables considered. Furthermore, we carried out stratified analyses to assess sources of heterogeneity across subgroups defined by adjustments for the important risk factors (Table 2). No significant differences were found between studies with and without adjustment for BMI, smoking, alcohol, vegetable and fruit consumption and upper gastrointestinal tract symptoms. The point estimates adjusted by BMI, smoking, alcohol and upper gastrointestinal tract symptoms tended to be higher than the unadjusted ones, however the point estimate adjusted by vegetable and fruit consumption was lower than the unadjusted one.

**Table 1:** Summary RRs of aspirin use and risk of gastric cancer, in strata of geographical region, study design, sample source, cancer site and helicobacter pylori infection.

Subgroups		No. of studies	No. of cases	RR(95%CI)	Statistical method	P-value for Heterogeneity^a^
All studies		15	6868	0.71(0.60–0.82)	Random	
Study design	Case-control	8	4437	0.67(0.54–0.81)	Random	0.331
	Cohort or RCT	7	2431	0.78(0.60–0.95)	Random	
Sample source	Hospital-based	4	1731	0.63(0.51–0.75)	Fixed	0.332
	Population-based	11	5137	0.72(0.59–0.86)	Random	
Geographical region	USA	9	3547	0.66(0.53–0.79)	Random	0.138
	Europe	5	2338	0.85(0.64–1.05)	Random	
	Asia	1	983	0.61(0.48–0.77)	Fixed	
Cancer site	Cardia	7	1222	0.81(0.60–1.03)	Random	0.098
	Non-cardia	8	2914	0.59(0.44–0.74)	Random	
Helicobacter pylori infection	Yes	2	366	0.49(0.28–0.70)	Fixed	0.148
	No	2	249	0.81(0.43–1.18)	Fixed	

RR, Relative risk; CI, confidence interval. ^a^ Two-sided z test was used to test heterogeneity of subgroups.

**Table 2:** Summary RRs of aspirin use and risk of gastric cancer, in strata of selected covariates.

Subgroups		No. of studies	No. of cases	RR(95%CI)	Statistical method	P-value for Heterogeneity^ a^
Adjustment for BMI	No	6	2585	0.60(0.43–0.77)	Random	0.134
	Yes	9	4283	0.78(0.64–0.92)	Random	
Adjustment for smoking	No	6	2585	0.60(0.43–0.77)	Random	0.134
	Yes	9	4283	0.78(0.64–0.92)	Random	
Adjustment for alcohol	No	11	4576	0.68(0.55–0.81)	Random	0.435
	Yes	4	2292	0.78(0.56–0.99)	Random	
Adjustment for UGI	No	12	4498	0.64(0.53–0.75)	Random	0.118
symptoms	Yes	3	2370	0.81(0.62–1.00)	Random	
Adjustment for vegetable and fruit consumption	No	12	6222	0.73(0.60–0.86)	Random	0.208
	Yes	3	646	0.59(0.43–0.75)	Random	

RR, Relative risk; CI, confidence interval; BMI, body mass index; UGI, upper gastrointestinal tract. **^a^** Two-sided z test was used to test heterogeneity of subgroups.

Galbraith plots showed that two lowest and two highest risk estimates in four studies [33]–[35], [37] were potential sources of heterogeneity, but the effect estimate excluding these heterogeneity results (RR  = 0.68, 95% CI 0.62–0.74) varied only slightly compared to the overall effect estimate.

### Frequency-risk and duration-risk relationships

**Figure 3:**
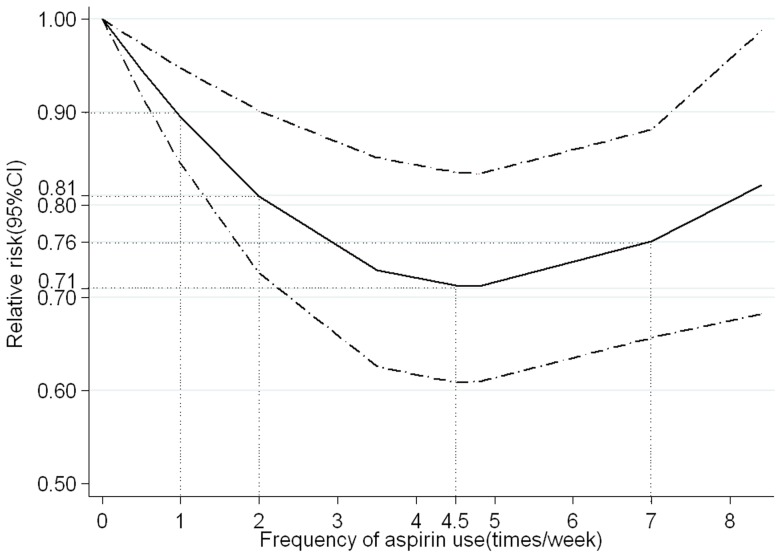
Association between frequency of aspirin use and risk of gastric cancer obtained by the restricted cubic spline regression model with 3 knots (0, 3.5, 8.4 times per week) and nonuse as reference. *P*_non-linearity_  = 0.005. Solid line represents the estimated relative risk and the dot-dashed lines represent the 95% confidence intervals. The dotted lines are used to explain the relative risk of gastric cancer for different frequency of aspirin use.

When the frequency of aspirin use was divided into two subgroups (<7 times/week and ≥7 times/week), there was no apparent trend with increasing frequency of aspirin use (RR  = 0.71, 95% CI 0.62–0.80, for <7 times/week users; RR  = 0.70, 95% CI 0.59–0.81, for ≥7 times/week users; Figure S1). However, the random-effect cubic spline model indicated a non-linear relation between frequency of aspirin use and gastric cancer risk (*P* for non-linearity = 0.005; Figure 3). The decreased risk of gastric cancer for the once per week aspirin user was 0.90 (95% CI 0.84–0.95), and there was a stronger risk reduction for the twice per week aspirin user(RR  = 0.81, 95% CI 0.73–0.90). However, for users of more than 4.5 times per week, there was no monotonically decreasing trend, and on the contrary, a monotonically increasing trend was observed (RR  = 0.71, 95% CI 0.61–0.84, for 4.5 times per week; RR  = 0.76, 95%CI 0.66–0.88, for 7 times per week; Table S5).

**Figure 4:**
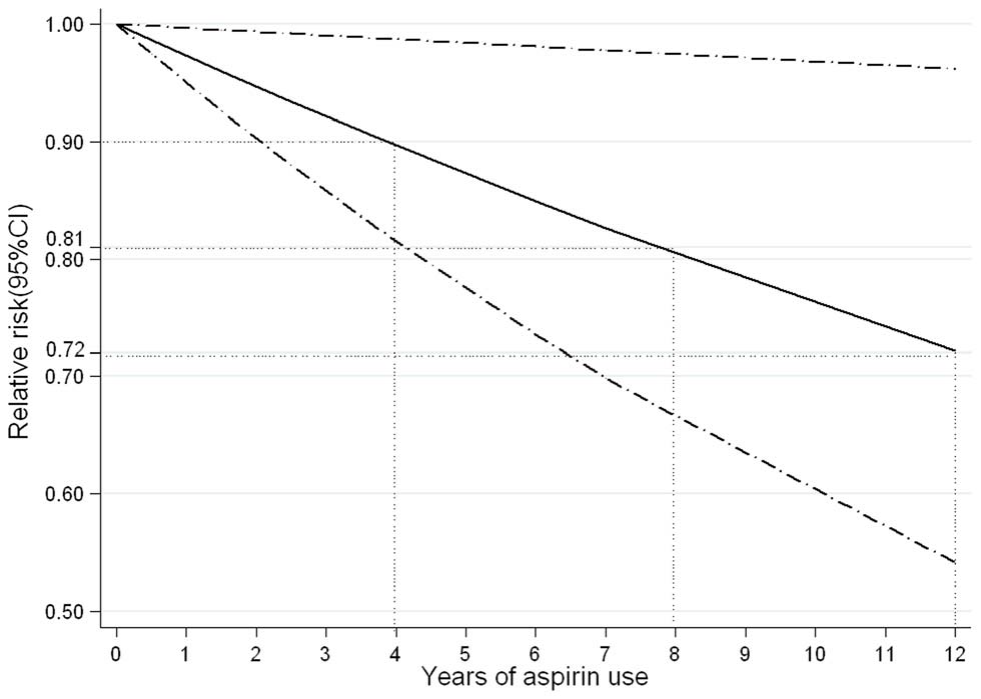
Association between years of aspirin use and risk of gastric cancer obtained by the linear regression model. *P*_linearity_  = 0.026. Solid line represents the estimated relative risk and the dot-dashed lines represent the 95% confidence intervals. The dotted lines are used to explain the relative risk of gastric cancer for different duration of aspirin use.

When the duration of aspirin use was divided into two subgroups (<5 years and ≥5 years), we observed a suggestive trend of decreasing risk of gastric cancer associated with increasing duration of aspirin use (RR  = 0.95, 95% CI 0.76–1.14, for <5 years; RR  = .67, 95% CI 0.56–0.79, for ≥5 years; Figure S2). In addition, a linear regression model was fitted (*P* for linear trend  = 0.026; Figure 4), since the non-linear relation between duration of aspirin use and gastric cancer risk had no significance in the cubic spline model (*P* for non-linearity  = 0.570; Figure S3). The risk of gastric cancer declined progressively as the duration of aspirin use increased. The risk of gastric cancer for 4 years of aspirin use was 0.90 (95% CI 0.82–0.99). There was a tendency towards stronger risk reduction for longer aspirin usage (RR  = 0.81, 95% CI 0.67–0.98, for 8 years; RR  = 0.72, 95% CI 0.54–0.96, for 12 years; Table S5).

### Publication bias

**Figure 5:**
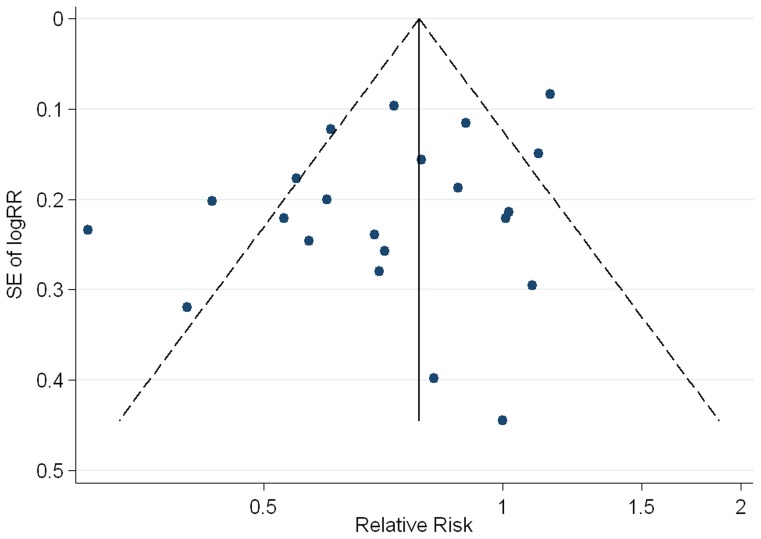
Begg's funnel plot with 95% confidence limits to detect publication bias. Each point represents a separate study for the indicated association.

Slight publication bias was observed from visual inspection of the funnel plot and from statistical tests (Begg's test P = 0.535; Egger's test P = 0.062, Figure 5). The RR estimate varied slightly after using the trim-and-fill method to adjust the potential publication bias (RR for trim-and-fill method  = 0.72, 95%CI 0.62–0.84), indicating that aspirin use was consistently associated with a decreased risk of gastric cancer.

## Discussion

Although there have been several meta-analyses on aspirin and gastric cancer, a few quantitative reviews reported an inverse association [6]–[10], while another meta-analysis found no significant association [11]. So we conducted an up-to-date meta-analysis in a larger number of cases and controls than previous reports to get a more credible conclusion, and at the same time we clarified the reasons for the different conclusions in previous studies. In addition, we built on past reviews by evaluating additional aspects of aspirin use, such as frequency and duration, and an important advantage of our pooled study is that we were able to explore if a threshold of effect exists between aspirin use and risk of gastric cancer.

Evidence from this updated meta-analysis of observational studies indicates a protective effect against gastric cancer, with the risk reduction for aspirin use being 29% (33% for case-control studies and 22% for cohort studies). This finding is consistent with several previous quantitative reviews [6]–[10], which report around 26%–33% reduction in the risk of gastric cancer for aspirin use. It was noteworthy that aspirin use may cause gastrointestinal bleeding and ulcer perforation [43], [44], and it is possible that patients with early symptoms of gastric cancer avoid using this drug. Additionally it is possible that aspirin increases the likelihood of being diagnosed with gastric cancer, as a result leading to an underestimate of the risk.

However, another meta-analysis of Yang [11] found no significant association between aspirin use and gastric cancer. After careful checking the inclusion and exclusion criteria, the overlap of studies and statistical analysis in Yang's study [11] and our study, we found that there are similarities and differences. First, as for the inclusion and exclusion criteria, both meta-analyses included case-control, cohort and RCT studies, but articles were searched from January 1980 to February 2013 in our study and from 1950 to January 2009 in Yang's study. Second, as for the overlap of studies included, we excluded two studies reported in Yang's study, since one study [45] with large standard error was not published in 1980–2013 but published in 1968 and the outcome of another study [46] is esophagogastric junctional adenocarcinoma rather than gastric cancer. In addition, four new studies [29], [31], [39], [41] had added in our study to give more reliable and valid results. Third, as for statistical analysis, we used the adjusted risk estimates to carry out meta-analysis, but the unadjusted risk estimates were used in Yang's study. Another difference is that the risk estimate of Lindblad-2005 [37] was 3.04 (95%CI 2.69–3.43) reported in Yang's study but this risk estimate in the original study was only 1.15(95%CI 0.98–1.36). So estimates from Yang's study may be less reliable and valid.

The most important question remains unclear, and that is the frequency-risk relationship between aspirin intake and gastric cancer. When aspirin use was divided into <7 times/week and ≥7 times/week, the interesting finding is that there was no apparent linear trend with increasing frequency of use (RR  = 0.71 for <7 times/week; RR  = 0.70 for ≥7 times/week). We suspect that there may be non-linear frequency-risk relation, so we performed a dose-response meta-analysis to clarify this hypothesis. We found that aspirin use is consistently associated with a decreased risk of gastric cancer, and even for the once a week user, a 10% reduction in gastric risk was observed. A more interesting and meaningful finding in our study is the existence of a threshold effect between frequency of aspirin use and risk of gastric cancer. For low-frequency (≤4.5 times per/week) aspirin intake, a monotonically decreasing trend was observed (RR  = 0.90 for once/week aspirin user; RR  = 0.81 for twice /week; RR  = 0.71 for 4.5 times/week). However, for high-frequency (>4.5 times/week) aspirin intake, an inverse and monotonically increasing trend was observed (RR  = 0.74 for 6 times/week; RR  = 0.76 for 7 times/week; RR  = 0.82 for 8 times/week). Therefore, the frequency threshold of aspirin use associated with the risk of gastric cancer is 4.5 times per week. Given the greater risk of bleeding complications caused by high-frequency use [47] as well as cost-effectiveness, the optimal frequency of aspirin for preventing gastric cancer may be within the range of 1–4.5 times per week, in which monotonically decreasing dose-response relationships and about 10%–29% reduction in risk of gastric cancer were observed. There was some evidence that 2–7 times per week aspirin use can reduce the incidence of colorectal cancer [24]. The overlapping range of aspirin use for protective effect suggests that regular aspirin use can simultaneously prevent gastric cancer and colorectal cancer.

It is also very important to clarify the duration-risk relationship between years of aspirin use and risk of gastric cancer. When the duration of aspirin use was divided into <5 years and ≥5 years, we observed a suggestive negative linear trend (RR  = 0.95 for <5 years; RR  = 0.67 for ≥5 years). In order to verify this trend, a duration-response meta-analysis using data on years of aspirin use was performed. An important finding is that a negative linear correlation between duration of aspirin use and gastric cancer risk was observed. There was a 10% reduced risk of gastric cancer for 4-year durations of aspirin use, and the decreased risk is almost double for 8-year durations and triple for 12-year durations. A more interesting finding is that the negative linear duration-risk relationship in this meta-analysis is similar with the recent meta-analysis of colorectal cancer which recommended at least 5 years of aspirin use for prevention of colorectal cancer [24]. The overlapping of protective effect suggests that long-term aspirin use can simultaneously prevent gastric cancer and colorectal cancer. However, in the pooled analysis of three RCTs of aspirin use for the prevention of cardiovascular diseases [42], a significant reduction of stomach cancer mortality was observed only after a long period of latency (RR  = 1.36, 95% CI 0.64–2.90, for 0–10 years' follow-up and RR  = 0.42, 95% CI 0.23–0.79, for 10–20 years' follow-up). The overlapping of protective effect suggests that long-term aspirin use can simultaneously prevent the incidence and mortality of gastric cancer. Therefore, long-term (at least 4 years) aspirin use is also recommended in prevention of gastric cancer.

When stratifying by cancer site and *H. pylori* infection, the risk estimates have no statistically significant difference between subgroups. However, aspirin use was associated with a significant reduction in the risk of non-cardia gastric cancer (RR  = 0.59, 95% CI 0.44–0.74) but not of cardia gastric cancer (RR  = 0.81, 95% CI 0.60–1.03) since only one of seven studies on cardia gastric cancer reported a significantly inverse association. This finding is consistent with the earlier meta-analyses [6], [8], [11]. Since cardia gastric cancer is different from non-cardia gastric cancer in both pathologic and clinical features [48], [49], it would not be surprising if the effect of aspirin differs in anatomical sites. Furthermore, the strong protective effect of aspirin among *H. pylori*-infected, but not among non-infected, subjects. Although the mechanisms underlying are not well understood, it has been suggested that aspirin may act by inhibiting one or more effects of *H. pylori*, which eventually lead to the development of gastric cancer [30].

In a subgroup analysis stratified by sample source, the risk estimate (RR  = 0.72) for population-based studies is closer to the overall estimate (RR  = 0.71) than the risk estimate for hospital-based studies (RR  = 0.63). Because the participants may not come from a single and well-defined population, hospital-based studies might be subject to selection bias and cause the distortion of results. However, such studies continue to be carried out since they are more convenient, expeditious and less expensive than population-based studies [7].

Since some heterogeneity was observed (I^2^  = 75.5%, P for heterogeneity = 0.000), we further explored the sources of heterogeneity by stratified analyses and Galbraith plots. Although no significant heterogeneity was found for any of the stratification variables considered, we found that the point estimates among Europe and cardia subgroups were higher than USA and non-cardia subgroup, and these differences will explain some heterogeneity. In addition, Galbraith plots showed that two lowest and two highest risk estimates in four studies [33]–[35], [37] were potential sources of heterogeneity. The characteristics of studies and definition of aspirin use will explain some heterogeneity, since the lowest risk estimates [33], [35] were from USA and non-cardia subgroups and one with highest risk estimates defined the reference group as no regular use (<2 pills per week) [34], and another with highest risk estimates defined aspirin use as any use [37].

Confounders are a major issue in observational studies. A biased association between an exposure and a disease can be inferred when the confounding factors are not controlled in either the study design and/or through statistical adjustment methods [50]. In order to avoid confounding by other major risk factors, including smoking, alcohol, overweight and obesity, low fruit and vegetable consumption and upper gastrointestinal tract symptoms, we used multivariate-adjusted risk estimates to perform this meta-analysis. Furthermore, no significant differences were found between the pooled RRs adjusted by these factors and the unadjusted ones, suggesting that residual confounding by smoking, BMI, fruit and vegetable consumption and gastrointestinal tract symptoms did not modify the association with aspirin.

There are several potential limitations to this meta-analysis. First, observational studies are susceptible to various biases because of their retrospective nature, so their test power is not as strong as that of experimental studies. Second, because of resource limitations, we did not attempt to search for unpublished studies, which could bring publication bias. However, visual inspection of funnel plot and statistical tests suggest only slight publication bias for studies. Additionally, the RR estimate varies only slightly after using the trim-and-fill method to adjust the meta-analysis estimates. Third, because of the lack of individual data, we could not adjust prevalence of aspirin use by factors that may influence aspirin use, such as the motivation for aspirin use [51]. Fourth, as in most meta-analyses, these results should be interpreted with caution because the definition of aspirin intake, lengths of follow-up, diagnosis method, and potential confounding factors adjusted were not uniform. Fifth, the limitation of our data is that no dosage information was collected in any of the studies. Inclusion of aspirin dose would have provided a better indicator of drug exposure than frequency and duration alone. Finally, although it is very meaningful to explore the relation between non-aspirin NSAIDs and gastric cancer risk, there are not sufficient data on non-aspirin NSAIDs to carry out dose-response meta-analyses.

In conclusion, the epidemiological evidence confirms that aspirin use is associated with reduced risk of gastric cancer. Such a favourable effect was observed in gastric non-cardia, *H. pylori*-infected, case-control and cohort and RCT studies, hospital-based and population-based population, American and Asian, and was not explained by smoking, alcohol, BMI and other relevant risk factors for gastric cancer. A completely novel finding in this meta-analysis is the existence of a threshold effect between frequency of aspirin intake and the risk of gastric cancer, suggesting that the recommended frequency for prevention of gastric cancer is 1–4.5 times per week. In addition, a linear duration-risk relationship was observed between years of aspirin use and gastric cancer risk, so long-term (≥4 years) consistent use of aspirin appears to be necessary to achieve effective protection. An open question for future research is whether a dose-response relationship exists considering other NSAIDs. Additionally, a large-scale randomized control trial in a population at high risk of gastric cancer is needed, in which aspirin's side effects should continually be monitored.

## Supporting Information

Figure S1 **Forest plot for the association between frequency of aspirin use and risk of gastric cancer, in strata of frequency of aspirin use.** The combined relative risk was achieved using fixed-effects model. Grey square represents relative risk in each study, with square size reflecting the study-specific weight and the 95% CI represented by horizontal bars. The diamond indicates summary risk estimate. Gastric NOS means that the location of the tumors within the stomach was not specified.
(TIF)

Figure S2 **Forest plot for the association between years of aspirin use and risk of gastric cancer, in strata of duration of aspirin use.** The combined relative risk was achieved using fixed-effects and random-effects models. Grey square represents relative risk in each study, with square size reflecting the study-specific weight and the 95% CI represented by horizontal bars. The diamond indicates summary risk estimate. Gastric NOS means that the location of the tumors within the stomach was not specified.
(TIF)

Figure S3 **Association between years of aspirin use and risk of gastric cancer obtained by the restricted cubic spline regression model with 3 knots (0, 2.5, 7 years) and nonuse as reference.** *P*_non-linearity_  = 0.570. Solid line represents the estimated relative risk and the dot-dashed lines represent the 95% confidence intervals. The dotted lines are used to explain the relative risk of gastric cancer for different duration of aspirin use (RR  = 0.92, 95% CI 0.80–1.06, for 4 years of aspirin use; RR  = 0.80, 95% CI 0.65–0.98, for 8 years; RR  = 0.67, 95% CI 0.46–0.99, for 12 years).
(TIF)

Table S1 **PRISMA checklist of this meta-analysis.**
(DOC)

Table S2 **Characteristics of studies included in the meta-analysis of aspirin use and gastric cancer.**
(DOC)

Table S3 **Epidemiological studies of frequency of aspirin use (times/week) and gastric cancer.**
(DOC)

Table S4 **Epidemiological studies of years of aspirin use and gastric cancer.**
(DOC)

Table S5 **RRs of aspirin use and gastric cancer estimated by dose-response models.**
(DOC)

Command S1 **The data structures and Stata commands in this Meta-analysis.**
(DOC)
