# Supplementary figures and images for: Frequency-Risk and Duration-Risk Relationships between Aspirin Use and Gastric Cancer: A Systematic Review and Meta-Analysis
Source: PLoS One. 2013 Jul 30;8(7):e71522. doi: 10.1371/journal.pone.0071522 (PMC3728206; doi:10.1371/journal.pone.0071522)

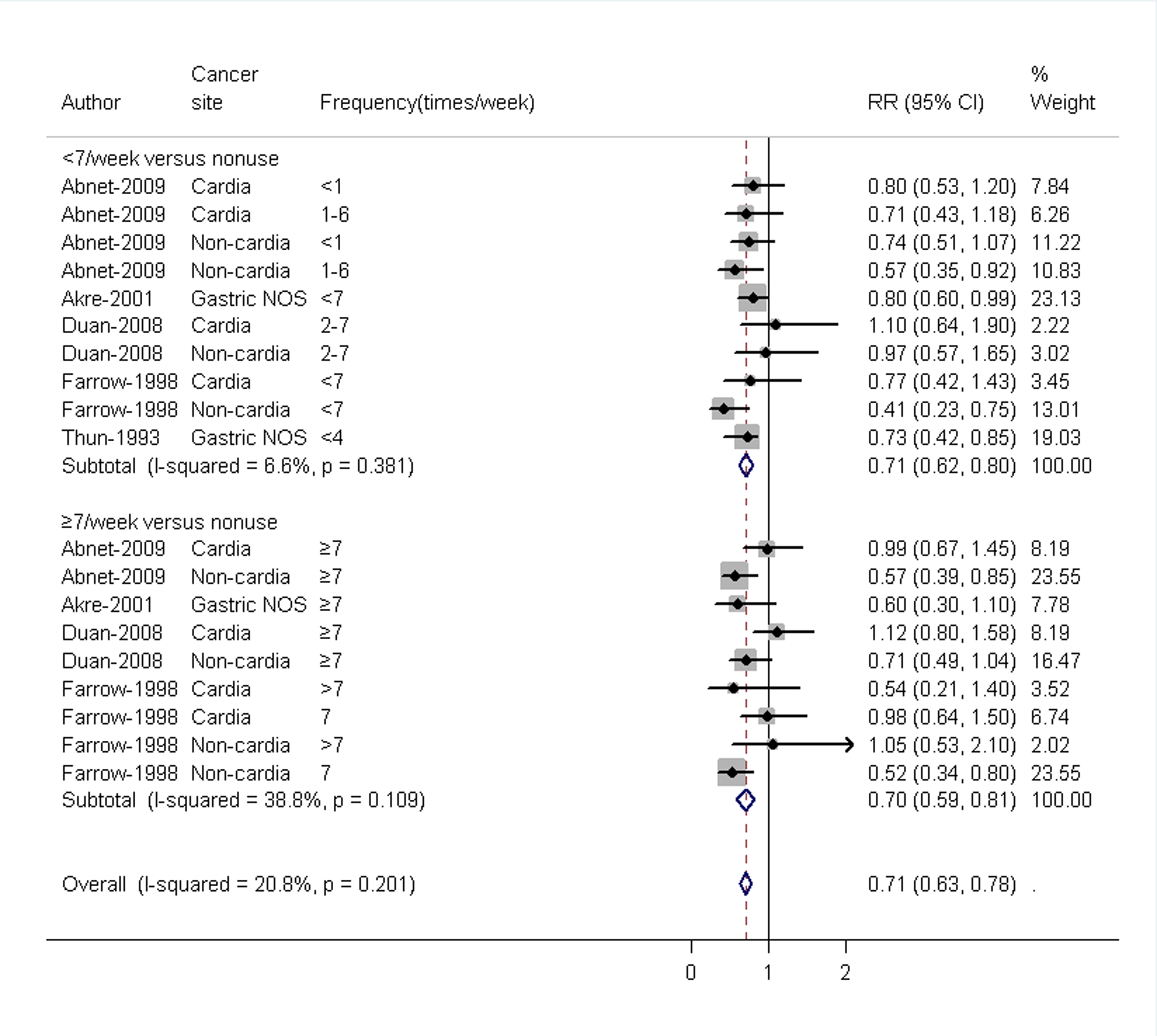

Supplement: Figure S1 — Forest plot for the association between frequency of aspirin use and risk of gastric cancer, in strata of frequency of aspirin use. The combined relative risk was achieved using fixed-effects model. Grey square represents relative risk in each study, with square size reflecting the study-specific weight and the 95% CI represented by horizontal bars. The diamond indicates summary risk estimate. Gastric NOS means that the location of the tumors within the stomach was not specified. (TIF) [file pone.0071522.s001.tif]

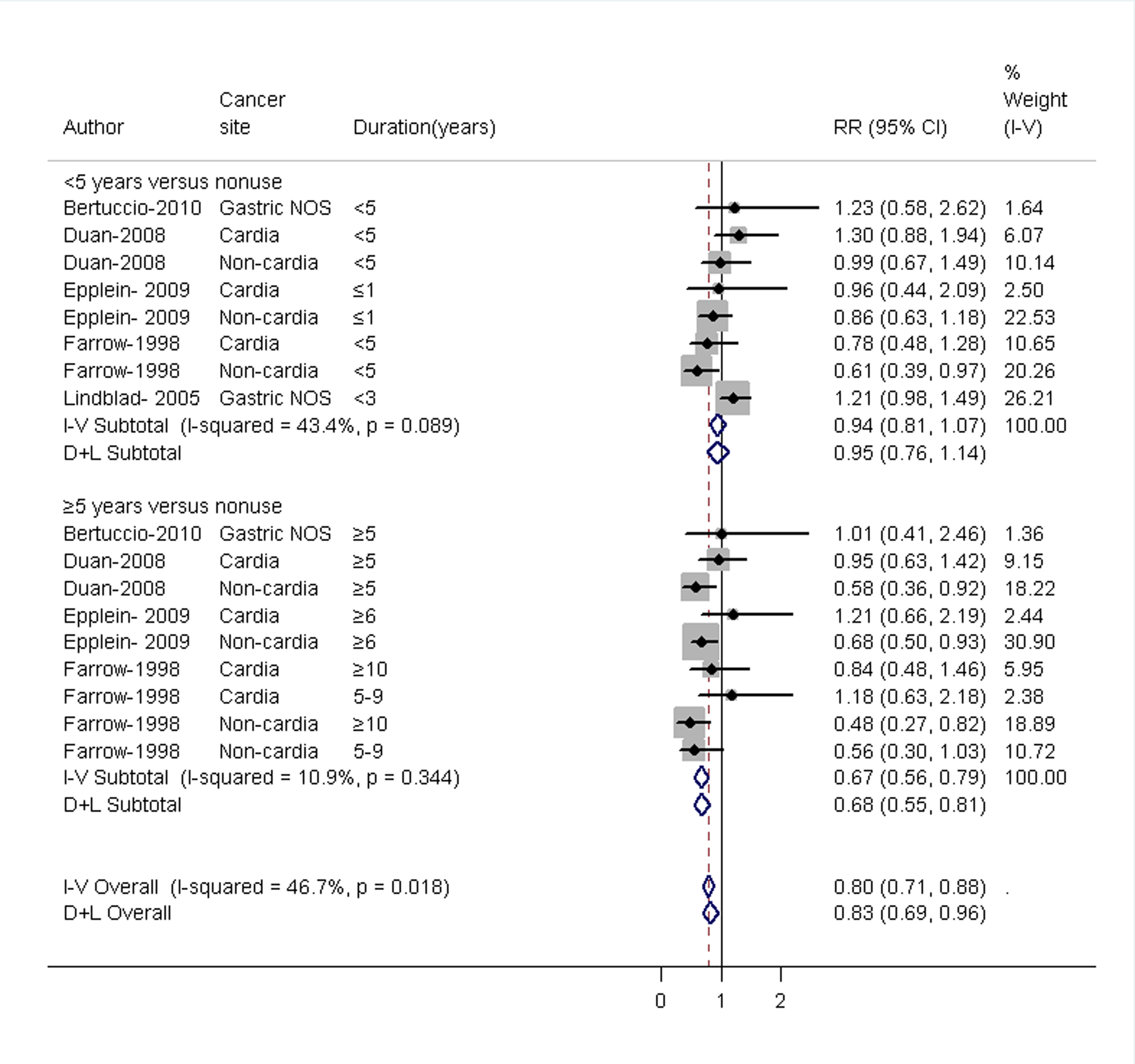

Supplement: Figure S2 — Forest plot for the association between years of aspirin use and risk of gastric cancer, in strata of duration of aspirin use. The combined relative risk was achieved using fixed-effects and random-effects models. Grey square represents relative risk in each study, with square size reflecting the study-specific weight and the 95% CI represented by horizontal bars. The diamond indicates summary risk estimate. Gastric NOS means that the location of the tumors within the stomach was not specified. (TIF) [file pone.0071522.s002.tif]

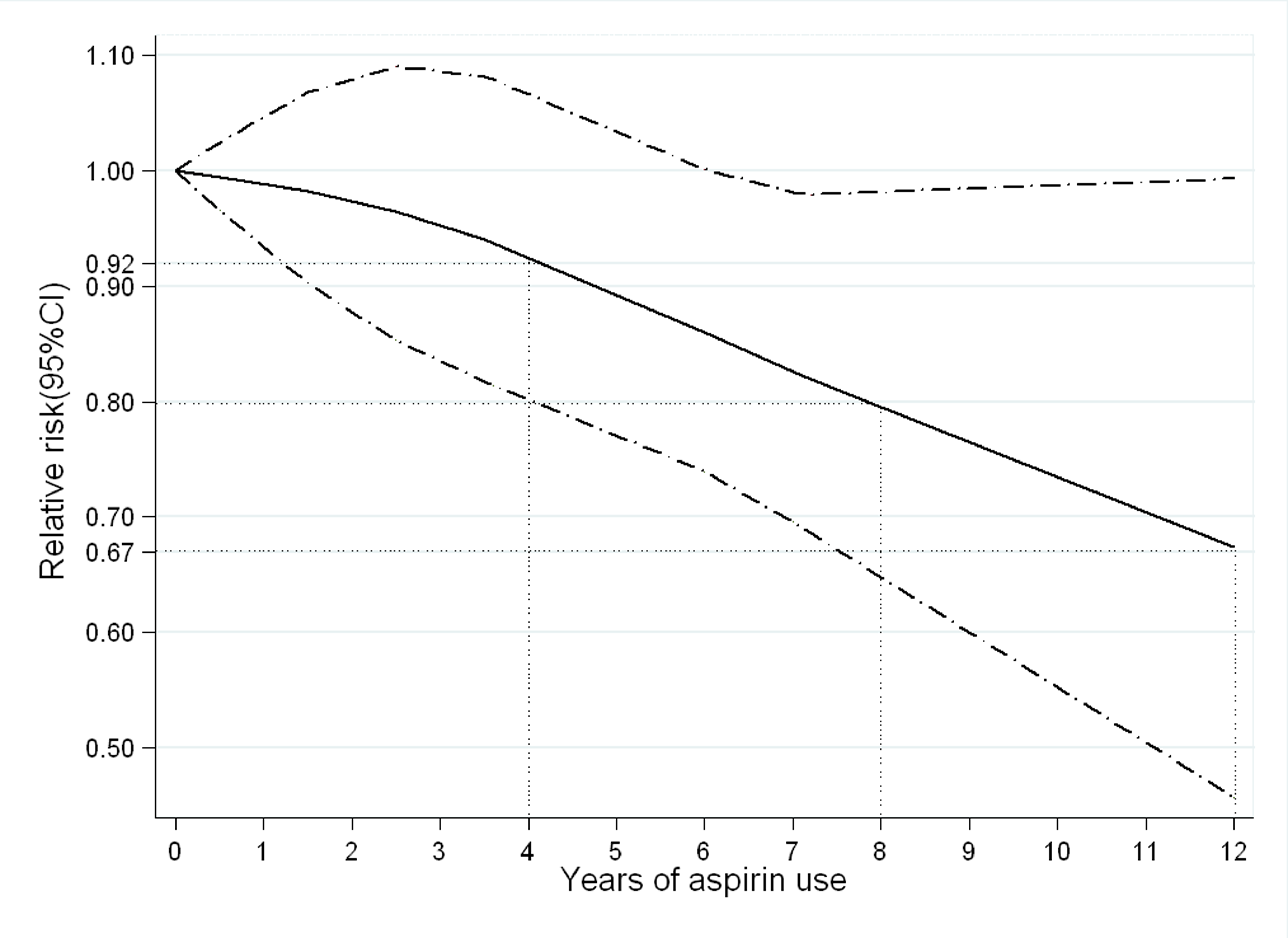

Supplement: Figure S3 — Association between years of aspirin use and risk of gastric cancer obtained by the restricted cubic spline regression model with 3 knots (0, 2.5, 7 years) and nonuse as reference. P non-linearity = 0.570. Solid line represents the estimated relative risk and the dot-dashed lines represent the 95% confidence intervals. The dotted lines are used to explain the relative risk of gastric cancer for different duration of aspirin use (RR = 0.92, 95% CI 0.80–1.06, for 4 years of aspirin use; RR = 0.80, 95% CI 0.65–0.98, for 8 years; RR = 0.67, 95% CI 0.46–0.99, for 12 years). (TIF) [file pone.0071522.s003.tif]
