# Supplementary material for: Frequency-Risk and Duration-Risk Relationships between Aspirin Use and Gastric Cancer: A Systematic Review and Meta-Analysis
Source: PLoS One. 2013 Jul 30;8(7):e71522. doi: 10.1371/journal.pone.0071522 (PMC3728206; doi:10.1371/journal.pone.0071522)
Supplement: Table S2 — Characteristics of studies included in the meta-analysis of aspirin use and gastric cancer. (DOC) [file pone.0071522.s005.doc]

**Table S2.** Characteristics of studies included in the meta-analysis of aspirin use and gastric cancer.

| **First author, year of**  **publication, reference** | **Study**  **design** | **Country** | **No. of case users/total cases** | **Definition of aspirin use (frequency, duration)** | **Diagnosis**  **method** | **Adjusted factors** |
| --- | --- | --- | --- | --- | --- | --- |
| Lee-2012[29] | HCC | Korea | 184/983 | any use | Cancer registry, pathology | Age, sex and statin use. |
| Zaridze-1999[30] | HCC | Russia | 48/448 | ≥ 2 per week, ≥6 months | Pathology | Age and education. |
| Bertuccio-2010[31] | HCC | Italy | 21/229 | ≥1 per week, ≥6 months | Pathology | Age, sex, education, BMI, smoking, and family history of gastric cancer. |
| Akre-2001[32] | PCC | Sweden | 170/567 | ≥1 per month | Cancer registry, pathology | Age, gender and socioeconomic status. |
| Fortuny-2007[33] | PCC | America | 43/496 | >7 prescriptions | Cancer registry | Age, sex, years of enrollment, race and drug classes. |
| Duan-2008[34] | PCC | America | 139/718 | ≥2 per week, ≥1 months | Cancer registry | Age, birthplace, BMI, education, sex, race, smoking, UGI history, and antacid use. |
| Figueroa- 2009[35] | PCC | America | 92/367 | ≥1 per week, ≥6 months | Pathology | Center, age, race, gender, smoking, gerd, proxy interview, and BMI. |
| Farrow-1998[36] | PCC | America | 159/629 | ≥1 per week, ≥6 months | Pathology | Age, gender, BMI, study center, smoking, income, and UGI symptoms. |
| Abnet-2009[10] | Cohort | America | 245/360 | ≥1 per week, ≥1 year | Cancer registry | Age, sex, smoking, alcohol, education, vegetable and fruit intake, BMI, energy intake, and physical activity. |
| Lindblad-2005[37] | Cohort | England | 519/1023 | any use | Database record, pathology | Sex, age, smoking, alcohol, BMI, calendar year, and UGI disorders. |
| Ratnasinghe-2004[38] | Cohort | America | 23/48 | any use | Cancer registry | BMI, sex, race, poverty index, education, and smoking. |
| Epplein-2009[39] | Cohort | California | 211/643 | ≥2 per week, ≥1 month | Cancer registry | Age, sex, ethnicity, smoking, BMI, and alcohol. |
| Thun-1993[40] | Cohort | America | 24/266 | ≥15 per month, ≥1 month | Cancer registry | Age, sex, race, BMI, smoking, alcohol, dietary fat, fruit/vegetable/grain intake, and family history of cancer. |
| Cook-2005 [41] | RCT | America | 10/20 | Every other day | Pathology | Age and vitamin E and beta carotene assignments. |
| Rothwell-2011 [42] | RCT | [United Kingdom](http://www.google.com.hk/url?q=http://en.wikipedia.org/wiki/United_Kingdom&sa=U&ei=dMCdUdL7CYqXkQWr0IHoAw&ved=0CBsQFjAA&usg=AFQjCNEhbylY71eAUtitOSbxul0aN3Rr0Q) | -/71 | Daily | Cancer registry | No mention |

HCC: hospital-based case-control; PCC: population-based case-control; RCT, randomized controlled trial; BMI: body mass index; UGI: upper gastrointestinal tract.
