# Supplementary material for: Frequency-Risk and Duration-Risk Relationships between Aspirin Use and Gastric Cancer: A Systematic Review and Meta-Analysis
Source: PLoS One. 2013 Jul 30;8(7):e71522. doi: 10.1371/journal.pone.0071522 (PMC3728206; doi:10.1371/journal.pone.0071522)
Supplement: Table S3 — Epidemiological studies of frequency of aspirin use (times/week) and gastric cancer. (DOC) [file pone.0071522.s006.doc]

**Table S3.** Epidemiological studies of frequency of aspirin use (times/week) and gastric cancer.

| **First author,**  **publication year** | **Study design** | **Cancer type** | **Frequency of aspirin ( times/week)** | **Frequency midpoint ( times/week)^a^** | **RR(95%CI)** |
| --- | --- | --- | --- | --- | --- |
| Farrow-1998^[36]^ | Case-control | Cardia | 0 | 0 | 1.00 |
|  |  | Cardia | <7 | 3.5 | 0.77(0.42-1.43) |
|  |  | Cardia | 7 | 7 | 0.98(0.64-1.50) |
|  |  | Cardia | >7 | 8.4 | 0.54(0.21-1.40) |
| Farrow-1998^[36]^ | Case-control | Non-cardia | 0 | 0 | 1.00 |
|  |  | Non-cardia | <7 | 3.5 | 0.41(0.23-0.75) |
|  |  | Non-cardia | 7 | 7 | 0.52(0.34-0.80) |
|  |  | Non-cardia | >7 | 8.4 | 1.05(0.53-2.10) |
| Akre-2001^[32]^ | Case-control | Gastric NOS | 0 | 0 | 1.00 |
|  |  | Gastric NOS | <7 | 3.5 | 0.80(0.60-0.99) |
|  |  | Gastric NOS | ≥7 | 8.4 | 0.60(0.30-1.10) |
| Duan-2008^[34]^ | Case-control | Cardia | <2 | 1 | 1.00 |
|  |  | Cardia | 2-7 | 4.5 | 1.10(0.64-1.90) |
|  |  | Cardia | ≥7 | 8.4 | 1.12(0.80-1.58) |
| Duan-2008^[34]^ | Case-control | Non-cardia | <2 | 1 | 1.00 |
|  |  | Non-cardia | 2-7 | 4.5 | 0.97(0.57-1.65) |
|  |  | Non-cardia | ≥7 | 8.4 | 0.71(0.49-1.04) |
| Thun-1993^[40]^ | cohort | Gastric NOS | 0 | 0 | 1.00 |
|  |  | Gastric NOS | <4 | 2 | 0.81(0.73-0.90) |
|  |  | Gastric NOS | ≥4 | 4.8 | 0.71(0.61-0.83) |
| Abnet-2009^[10]^ | cohort | Cardia | 0 | 0 | 1.00 |
|  |  | Cardia | <1 | 0.5 | 0.80(0.53-1.20) |
|  |  | Cardia | 1-6 | 3.5 | 0.71(0.43-1.18) |
|  |  | Cardia | ≥7 | 8.4 | 0.99(0.67-1.45) |
| Abnet-2009^[10]^ | cohort | Non-cardia | 0 | 0 | 1.00 |
|  |  | Non-cardia | <1 | 0.5 | 0.74(0.51-1.07) |
|  |  | Non-cardia | 1-6 | 3.5 | 0.57(0.35-0.92) |
|  |  | Non-cardia | ≥7 | 8.4 | 0.57(0.39-0.85) |

Gastric NOS, the location of the tumours within the stomach was not specified; RR, Relative risk. ^a^ When intervals of aspirin categories were reported, the midpoint of the interval was chosen; For the open-ended upper interval, we used 1.2-fold its lower limit.
