# Supplementary material for: Frequency-Risk and Duration-Risk Relationships between Aspirin Use and Gastric Cancer: A Systematic Review and Meta-Analysis
Source: PLoS One. 2013 Jul 30;8(7):e71522. doi: 10.1371/journal.pone.0071522 (PMC3728206; doi:10.1371/journal.pone.0071522)
Supplement: Table S4 — Epidemiological studies of years of aspirin use and gastric cancer. (DOC) [file pone.0071522.s007.doc]

**Table S4.** Epidemiological studies of years of aspirin use and gastric cancer.

| **First author,**  **publication year** | **Study design** | **Cancer type** | **Years of**  **aspirin** | **Years**  **midpoint ^a^** | **RR(95%CI)** |
| --- | --- | --- | --- | --- | --- |
| Farrow-1998^[36]^ | Case-control | Cardia | 0 | 0 | 1.00 |
|  |  | Cardia | <5 | 2.5 | 0.78(0.48-1.28) |
|  |  | Cardia | 5-9 | 7 | 1.18(0.63-2.18) |
|  |  | Cardia | ≥10 | 12 | 0.84(0.48-1.46) |
| Farrow-1998^[36]^ | Case-control | Non-cardia | 0 | 0 | 1.00 |
|  |  | Non-cardia | <5 | 2.5 | 0.61(0.39-0.97) |
|  |  | Non-cardia | 5-9 | 7 | 0.56(0.30-1.03) |
|  |  | Non-cardia | ≥10 | 12 | 0.48(0.27-0.82) |
| Lindblad- 2005^[37]^ | Cohort | Gastric NOS | 0 | 0 | 1.00 |
|  |  | Gastric NOS | <3 | 1.5 | 1.21 (0.98-1.49) |
|  |  | Gastric NOS | ≥3 | 3.6 | 1.09(0.82-1.45) |
| Duan-2008^[34]^ | Case-control | Cardia | 0 | 0 | 1.00 |
|  |  | Cardia | <5 | 2.5 | 1.30(0.88-1.94) |
|  |  | Cardia | ≥5 | 6 | 0.95(0.63-1.42) |
| Duan-2008^[34]^ | Case-control | Non-cardia | 0 | 0 | 1.00 |
|  |  | Non-cardia | <5 | 2.5 | 0.99(0.67-1.49) |
|  |  | Non-cardia | ≥5 | 6 | 0.58(0.36-0.92) |
| Epplein- 2009^[39]^ | Cohort | Cardia | 0 | 0 | 1.00 |
|  |  | Cardia | ≤1 | 0.5 | 0.96(0.44-2.09) |
|  |  | Cardia | 2-5 | 3.5 | 1.11(0.59-2.08) |
|  |  | Cardia | ≥6 | 7.2 | 1.21(0.66-2.19) |
| Epplein- 2009^[39]^ | Cohort | Non-cardia | 0 | 0 | 1.00 |
|  |  | Non-cardia | ≤1 | 0.5 | 0.86(0.63-1.18) |
|  |  | Non-cardia | 2-5 | 3.5 | 0.69(0.51-0.93) |
|  |  | Non-cardia | ≥6 | 7.2 | 0.68(0.50-0.93) |
| Bertuccio-2010^[31]^ | Case-control | Gastric NOS | 0 | 0 | 1.00 |
|  |  | Gastric NOS | <5 | 2.5 | 1.23 (0.58-2.62) |
|  |  | Gastric NOS | ≥5 | 6 | 1.01(0.41-2.46) |

Gastric NOS, the location of the tumours within the stomach was not specified; RR, Relative risk. ^a^ When intervals of aspirin categories were reported, the midpoint of the interval was chosen; For the open-ended upper interval, we used 1.2-fold its lower limit.
