# Supplementary material for: Frequency-Risk and Duration-Risk Relationships between Aspirin Use and Gastric Cancer: A Systematic Review and Meta-Analysis
Source: PLoS One. 2013 Jul 30;8(7):e71522. doi: 10.1371/journal.pone.0071522 (PMC3728206; doi:10.1371/journal.pone.0071522)
Supplement: Table S5 — RRs of aspirin use and gastric cancer estimated by dose-response models. (DOC) [file pone.0071522.s008.doc]

**Table S5.** RRs of aspirin use and gastric cancer estimated by dose-response models.

| **Exposure** | **RR(95%CI)** |  | **Exposure** | **RR(95%CI)** |
| --- | --- | --- | --- | --- |
| Frequency of use |  |  | Duration of use |  |
| 0 /week | 1.00 |  | 0year | 1.00 |
| 1 /week | 0.90(0.84-0.95) |  | 2 years | 0.95(0.90-0.99) |
| 2 /week | 0.81(0.73-0.90) |  | 4years | 0.90(0.82-0.99) |
| 3 /week | 0.76(0.66-0.87) |  | 5years | 0.87(0.78-0.98) |
| 4.5 /week | 0.71(0.61-0.84) |  | 6years | 0.85(0.74-0.98) |
| 6 /week | 0.74(0.64-0.86) |  | 8 years | 0.81(0.67-0.98) |
| 7/week | 0.76(0.66-0.88) |  | 10years | 0.76(0.60-0.90) |
| 8/week | 0.80 (0.68-0.96) |  | 12years | 0.72(0.54-0.96) |

RR, Relative risk.
