# Supplementary material for: Frequency-Risk and Duration-Risk Relationships between Aspirin Use and Gastric Cancer: A Systematic Review and Meta-Analysis
Source: PLoS One. 2013 Jul 30;8(7):e71522. doi: 10.1371/journal.pone.0071522 (PMC3728206; doi:10.1371/journal.pone.0071522)
Supplement: Command S1 — The data structures and Stata commands in this Meta-analysis. (DOC) [file pone.0071522.s009.doc]

Analysis the association between aspirin use (ever use versus nonuse) and gastric cancer

1) Data Structures

| **no** | **author** | **design** | **site** | **case** | **RR** | **RRL** | **RRU** |
| --- | --- | --- | --- | --- | --- | --- | --- |
| 1 | Abnet-2009 | Cohort | Cardia | 130/178 | 0.71 | 0.43 | 1.18 |
| 1 | Abnet-2009 | Cohort | Non-cardia | 115/182 | 0.57 | 0.35 | 0.92 |
| 2 | Akre-2001 | Case-control | Cardia | 25/90 | 0.70 | 0.40 | 1.20 |
| 2 | Akre-2001 | Case-control | Non-cardia | 145/477 | 0.90 | 0.70 | 1.10 |
| 3 | … | … | … | … | … | … | … |

2) Variable Description

**no**: the individual number of each study

**author**: contains first author and year of publication

**design**: denotes study design

**site**: denotes the site of gastric cancer

**case**: contains cases of aspirin user and total cases

**RR**: denotes all risk estimates.

**RRL**: denotes the lower interval of 95% confidence intervals of RR

**RRU**: denotes the upper interval of 95% confidence intervals of RR

3) Meta-analyses using commands in Stata software

// use the -metan- command for fixed-effect meta-analysis; lcols() define columns of additional data to the left of the graph.

metan RR RRL RRU, fixed lcols(author design site case )

// use the -metan- command for random-effect meta-analysis

metan RR RRL RRU, random lcols(author design site case )

// use the -metan- command for subgroup analysis stratified by study design; second() specifies that a second analysis may be performed using another method; by( ) specifies that the meta-analysis is to be stratified according to the variable declared.

metan RR RRL RRU, second(random) lcols(author site) by(design )

// get logRR and standard errors of logRR

gen logRR=log(RR)

gen selogRR=(log(RRU)-log(RRL))/(2*invnormal(0.975))

// use the - galbr - command to fit Galbraith plots; id( ) supplied any variable, which is used to label the studies.

galbr logRR selogRR, id(no)

// use the –metafunnel- command to fit funnel plots

metafunnel logRR selogRR

// use the –metabias- command for Begg’s test for funnel-plot asymmetry in meta-analysis

metabias logRR selogRR , begg

// use the –metabias- command for Egger’s test for funnel-plot asymmetry in meta-analysis

metabias logRR selogRR ,egger

// use the –metatrim – command to adjust the risk estimates by the trim-and-fill method

metatrim logRR selogRR

Analysis the dose-response relation between frequency of aspirin use and gastric cancer

1) Data Structures

| **author** | **no** | **design** | **n** | **case** | **RR** | **RRL** | **RRU** | **Frequency** |
| --- | --- | --- | --- | --- | --- | --- | --- | --- |
| Farrow-1998 | 1 | 1 | 649 | 178 | 1 | 1 | 1 | 0 |
| Farrow-1998 | 1 | 1 | 83 | 18 | 0.77 | 0.42 | 1.43 | 3.5 |
| Farrow-1998 | 1 | 1 | 171 | 48 | 0.98 | 0.64 | 1.5 | 7 |
| Farrow-1998 | 1 | 1 | 37 | 9 | 0.54 | 0.21 | 1.4 | 8.4 |
| Akre-2001 | 2 | 1 | 930 | 624 | 1 | 1 | 1 | 0 |
| Akre-2001 | 2 | 1 | 336 | 247 | 0.80 | 0.60 | 0.99 | 3.5 |
| Akre-2001 | 2 | 1 | 61 | 47 | 0.60 | 0.30 | 1.1 | 8.4 |
| … | … | … | … | … | … | … | … | … |

2) Variable Description

**author**: contains first author and year of publication

**no**: the individual number of each study

**design**: denotes study design, and must take value 1 for case-control, 2 for incidence-rate, and 3 for cumulative incidence study.

**n**: is the number of subjects (controls plus cases) for case-control data; or the total person-time for incidence rate data; or the total number of persons (cases plus noncases) for cumulative incidence data.

**case**: contains the number of cases at each exposure level of aspirin use.

**RR**: denotes all risk estimates.

**RRL**: denotes the lower interval of 95% confidence intervals of RR

**RRU:** denotes the upper interval of 95% confidence intervals of RR

**Frequency**: denotes the frequency of aspirin use at each exposure level. When intervals of aspirin categories were reported, the midpoint of the interval was chosen. For the open-ended upper interval, we used 1.2-fold its lower limit.

3) Dose-response meta-analyses using commands in Stata software

// get logRR and standard errors of logRR

gen logRR=log(RR)

gen selogRR=(log(RRU)-log(RRL))/(2*invnormal(0.975))

// use the –glst- command to fit linear dose-response model; se( ) specifies the standard error of log relative risks; cov( ) specifies variables containing the information required to fit the covariances among correlated log realtive risks; pfirst( ) specifies the pool-first method with multiple summarized studies; random specifies the iterative generalized least squares method to estimate a random-effect metaregression model.

glst logRR frequency , se(selogRR) cov(n case) pfirst(no design) eform random

// use the –rc_spline - command to define 2 spline covariates associated with 3 knots

rc_spline frequency, nknots(3)

// use the –glst- command to fit random-effect cubic spline model by adding the spline covariates in the model

glst logRR _S* , se(selogRR) cov(n case) pfirst(no design) eform random

Analysis the dose-response relation between duration of aspirin use and gastric cancer

The analysis process on duration of aspirin use is the same as that of frequency data reported above.
